# Microstate‐Specific Cross‐Frequency Coupling Networks: A Spatiotemporal Framework for MCI Identification

**DOI:** 10.1002/cns.71036

**Published:** 2026-07-28

**Authors:** Feifei Yin, Luyang Liu, Lijie Gao, Chun Ji, Lili He, Aihua Lian, Xiuchun Yang, Xiaole Wang, Lida Chen, Kaixin Hui, Pengxiang Zuo, Junfeng Gao

**Affiliations:** ^1^ School of Medicine Shihezi University Shihezi China; ^2^ Key Laboratory of Cognitive Science of State Ethnic Affairs Commission, College of Biomedical Engineering South‐Central Minzu University Wuhan China; ^3^ Shihezi City People's Hospital Shihezi China

**Keywords:** brain networks, cross‐frequency coupling, EEG microstates, machine learning, mild cognitive impairment, phase synchronization

## Abstract

**Aims:**

This study integrated EEG microstates with cross‐frequency coupling networks to develop a novel spatiotemporal framework for the early identification of mild cognitive impairment (MCI).

**Methods:**

Resting‐state EEG was recorded from 22 patients with MCI and 22 age‐matched healthy controls. Topographic clustering was used to derive four canonical microstates (A–D). Source reconstruction was then performed to extract time series from seven resting‐state networks. For each microstate, a microstate‐specific cross‐frequency coupling network was constructed by computing n:m phase synchronization across the delta, theta, alpha, and beta bands among these networks. Classification was performed using Maximum Relevance Minimum Redundancy (mRMR) feature selection and a nested cross‐validated CatBoost classifier.

**Results:**

Significant disruptions in cross‐frequency phase synchronization were observed within microstates A, C, and D among patients with MCI. The classification model demonstrated its highest performance within microstate D, achieving a balanced accuracy of 84.4%. The features most effective in discriminating between groups primarily involved the limbic network in the theta band, the frontoparietal and dorsal attention networks in the alpha band, and the sensorimotor network in the delta band.

**Conclusion:**

MCI is associated with microstate‐dependent impairments in cross‐frequency synchronization, which suggests compromised spatiotemporal integration across large‐scale brain networks. The microstate‐specific cross‐frequency coupling network framework proposed offers a novel and physiologically interpretable EEG biomarker for the early diagnosis of MCI.

## Graphical abstract

Based on resting‐state EEG from older adults with mild cognitive impairment (MCI), this study introduces a novel Microstate‐specific Cross‐Frequency Coupling Network (MCFCN) framework by integrating EEG microstate analysis with cross‐frequency phase synchronization. This framework first reveals distinct patterns of phase synchronization disruption specifically within microstates A, C, and D in MCI. A classification model utilizing these abnormal network features achieves a balanced accuracy of 84.5% in discriminating MCI from healthy controls, offering a novel and interpretable EEG‐based biomarker for early MCI identification.

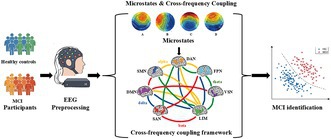

## 1 Introduction

Alzheimer's disease (AD) is the most prevalent type of dementia with a progressive neurodegenerative course [1]. Mild cognitive impairment (MCI) is an intermediate stage between normal aging and dementia [2]. Individuals with MCI face a substantially elevated risk of progressing to AD compared to their cognitively normal peers [3].

Early detection of MCI is key for predicting progression to AD [4]. Widely used diagnostic guidelines indicate that an MCI diagnosis primarily hinges on objectively verified cognitive decline, particularly in memory, as confirmed by neuropsychological testing. Importantly, individuals with MCI generally maintain their overall cognitive function and capacity for daily activities [5]. Current diagnostic approaches, however, present several limitations. Neuropsychological assessments are susceptible to confounding factors like education level, cultural background, and testing conditions, which introduce subjectivity and variability. Moreover, given the clinical heterogeneity of MCI, behavioral measures alone offer limited accuracy for early detection and progression prediction. Therefore, identifying objective, stable, and reproducible biomarkers is critically important for the early screening, risk stratification, and intervention monitoring of MCI.

No disease‐modifying therapy has been shown to prevent the conversion from MCI to AD; current pharmacological and antibody‐based interventions offer only limited slowing without reversing underlying pathology. Integrating multimodal biomarkers with multi‐target therapies and multidomain lifestyle approaches may enhance the prevention of clinical conversion [6, 7]. Intervention at the MCI stage may therefore lead to improved outcomes, necessitating sensitive early biomarkers to identify high‐risk individuals and facilitate targeted therapy development and evaluation.

Neuroimaging advancements have significantly deepened our understanding of alterations in brain structure and functional integrity in MCI. Resting‐state fMRI, for instance, reveals disrupted networks, characterized by diminished connectivity within the default mode and executive control networks. Furthermore, abnormal activity in the hippocampus and posterior cingulate cortex has been associated with accelerated decline in memory and executive decline [8]. Structural MRI reveals atrophy in the entorhinal cortex and hippocampus, while DTI indicates reduced fractional anisotropy, consistent with white‐matter damage. These structural and microstructural changes, combined with functional disconnection, collectively contribute to cognitive impairment [9]. Combining multimodal MRI with Aβ and tau PET imaging yields biomarkers that more accurately predict disease progression and define disease subtypes. Patients with stable or improving conditions often demonstrate preserved hippocampal volume and robust DMN connectivity. Conversely, patients experiencing decline typically exhibit prefrontal and temporal atrophy alongside increased Aβ burden, which may suggest early functional compensation [10]. Early tau accumulation disrupts the entorhinal–hippocampal circuit, and this disruption, coupled with white matter hyperintensities related to small‐vessel disease, exacerbates memory and executive deficits [11]. Overall, multimodal imaging enhances the detection of subclinical pathology and improves diagnostic specificity in MCI.

Unlike neuroimaging, EEG provides millisecond temporal resolution, which allows for real‐time assessment of synaptic‐level neural activity and the dynamic mechanisms underlying cognitive impairment [12]. Because synaptic injury is central to AD pathology [13], EEG is well suited to detect related functional abnormalities. It is also noninvasive, low‐cost, repeatable, and practical for longitudinal clinical monitoring [14]. Studies have reported reduced alpha (8–13 Hz) and beta (13–30 Hz) connectivity in individuals with MCI. This reduction has been linked to visuospatial memory deficits and may indicate inefficient attention and spatial processing [15]. Decreased delta/theta synchronization further indicates synaptic dysfunction and impaired neural coordination in amnestic MCI, which may disrupt information integration and memory retrieval [16].

Conventional frequency‐domain EEG analyses often miss interactions across bands. Cross‐frequency coupling (CFC), including phase–amplitude coupling (PAC) and cross‐frequency phase synchronization (CFS), helps explain coordination across large‐scale networks. CFC disruptions are common in AD and may impair network integration and worsen cognition [17]. CFS reflects stable n:m phase relationships across frequencies and is linked to consistent spiking activity [18]. It is quantified by the n:m phase synchronization index (*n:m* PSI), which rescales phase time series to match instantaneous frequencies; phase‐locking value quantifies the 1:1 (same‐frequency) synchronization case [19]. Although research on CFC in AD and MCI has expanded, the majority of studies have concentrated on PAC, leaving n:m PSI largely unexplored. A systematic evaluation of n:m PSI in MCI could elucidate early oscillatory dysfunction and facilitate the development of novel EEG biomarkers for diagnosis.

Microstate analysis is a technique that tracks sequences of whole‐brain EEG topographies, conceptualizing brain activity as a series of quasi‐stable states characterized by consistent topographical maps. The temporal dynamics of these microstates provide an index of coordination across large‐scale cortical networks and long‐range synchrony [20]. Resting EEG microstates are typically clustered into four classes (A–D), which have been putatively associated with the default mode, visual, dorsal attention, and salience networks [21]. The observed instability in these dynamics may signify impaired integration across default mode, salience, and occipito‐parietal circuits. This fragmentation within the network could accelerate cognitive decline and increase the risk of conversion to AD [22].

Few studies have combined cross‐frequency coupling with microstate analysis, resulting in an incomplete spatiotemporal–spectral understanding of MCI. Given that cross‐frequency coordination is physiologically linked to microstate stability, their combined application may offer a more comprehensive insight into network dysfunction. Here, we integrate canonical microstates to establish stable topographic windows and subsequently compute state‐specific cross‐frequency coupling networks for comparison between healthy controls and individuals with MCI. We then employ feature selection and machine learning to pinpoint the most discriminative biomarkers. This Microstate‐specific Cross‐Frequency Coupling Network (MCFCN) framework presents an electrophysiological methodology for the early detection and risk stratification of MCI.

## 2 Materials and Methods

### 2.1 Participants

We recruited 49 participants from a tertiary hospital in Shihezi, Xinjiang. All provided written informed consent and underwent neuropsychological testing, blood tests, and MRI to exclude other neurological or major systemic disorders. The study was approved by the Ethics Committee of the First Affiliated Hospital of Shihezi University (Approval No.: KJ2025‐332‐02). Resting‐state, eyes‐closed EEG was recorded. Five participants (2 MCI, 3 controls) were excluded for excessive artifacts, leaving 22 healthy controls and 22 MCI patients. Global cognition was assessed with the Montreal Cognitive Assessment (MoCA). Participant characteristics are shown in Table 1.

**TABLE 1:** Demographic and clinical characteristics of the study sample.

Variables	HC group (*n* = 22)	MCI group (*n* = 22)	*p*
Age, years (M ± SD)	56.27 ± 3.86	58.5 ± 4.93	0.103^a^
Sex (Female/Male)	14/8	10/12	0.087^b^
Education, years (*M* ± SD)	11.05 ± 1.94	11.32 ± 2.06	0.653^a^
MOCA (*M* ± SD)	25.41 ± 1.68	19.55 ± 3.16	< 0.001^a^

Abbreviations: HC, healthy controls; MCI, mild cognitive impairment; MoCA, Montreal Cognitive Assessment.

^a^ Two‐tailed independent sample *t*‐test.

^b^ A chi‐square test.

### 2.2 Data Acquisition and Preprocessing

Resting‐state EEG was recorded with a NeuroScan SynAmps2 system (NeuroScan, Charlotte, NC, USA) using a 64‐channel Ag/AgCl cap positioned by the international 10–20 system. The ground electrode was positioned between Fpz and Fz, and the reference electrode between Cz and CPz. Data were acquired at a sampling rate of 1000 Hz, with electrode impedances maintained below 10 kΩ. The acquisition took place during a 5‐min eyes‐closed session, where participants were instructed to sit comfortably, remain relaxed, awake, and still. Continuous monitoring was employed throughout the session to prevent drowsiness. Subsequent preprocessing of the data was carried out using EEGLAB [23]: a 50 Hz notch and a 1–30 Hz bandpass filters were applied, data were downsampled to 500 Hz, and signals were re‐referenced to the average mastoids (TP9/TP10). Independent component analysis was subsequently employed. The Adjust plug‐in facilitated the identification and removal of components associated with eye blinks and muscle artifacts.

### 2.3 Microstate Analysis

Microstate analysis was performed on 5‐min resting EEG data from 22 healthy controls and 22 patients with MCI using Microstate Lab. The analysis focused exclusively on the four canonical microstate classes (A–D). This restriction is consistent with common practice in clinical EEG and reflects their widespread recognition as the most stable and consistently reported set across studies [24]. Microstate templates were derived using K‐means clustering to capture the dominant spatiotemporal patterns [25]. The clustering procedure is outlined below. Initially, k‐means clustering was performed at the single‐subject level. Global field power (*GFP*), which is the standard deviation of voltage across electrodes at each time point, was calculated to quantify overall EEG strength. Topographic maps corresponding to *GFP* peaks; peaks were then chosen as stable “individual maps” for clustering, owing to their inherently higher signal‐to‐noise ratio [26]. *GFP* is defined as:

(1) $$\mathrm{GFP}=\sqrt{\frac{\sum_{i=1}^{N}{\left(u_{i}-\bar{u}\right)}^{2}}{N}}$$

where $u_{i}$ is the voltage at electrode i, $\bar{u}$ is the mean voltage across all electrodes, and N is the total number of electrodes.

Group‐level clustering was performed using the k‐means algorithm. We computed the spatial correlation between candidate templates and individual maps. The global explained variance (*GEV*) was used as a quantitative evaluation metric until the template topography achieved maximal spatial correlation with individual maps and stabilized. *GEV* is defined as:

(2) $$\mathrm{GEV}=\frac{\sum_{t=1}^{T}{\left[\mathrm{GFP}\left(t\right)\cdot \text{Corr}\left(V\left(t\right),M_{n}\right)\right]}^{2}}{\sum_{t=1}^{T}{\left[\mathrm{GFP}\left(t\right)\right]}^{2}}$$

where $\mathrm{GFP}\left(t\right)$ is the global field power at time t; $\text{Corr}\left(V\left(t\right),M_{n}\right)$ is the spatial correlation between the topography at t and the template map n; and T is the total number of time points.

To develop individual microstate sequences, group‐level templates were back‐fitted to each participant's continuous EEG by assigning each time point to the class with the highest spatial correlation. A 20 ms smoothing threshold was applied to merge brief segments and reassign them to adjacent microstates exhibiting higher correlation [27].

### 2.4 Source Reconstruction

**FIGURE 1:**
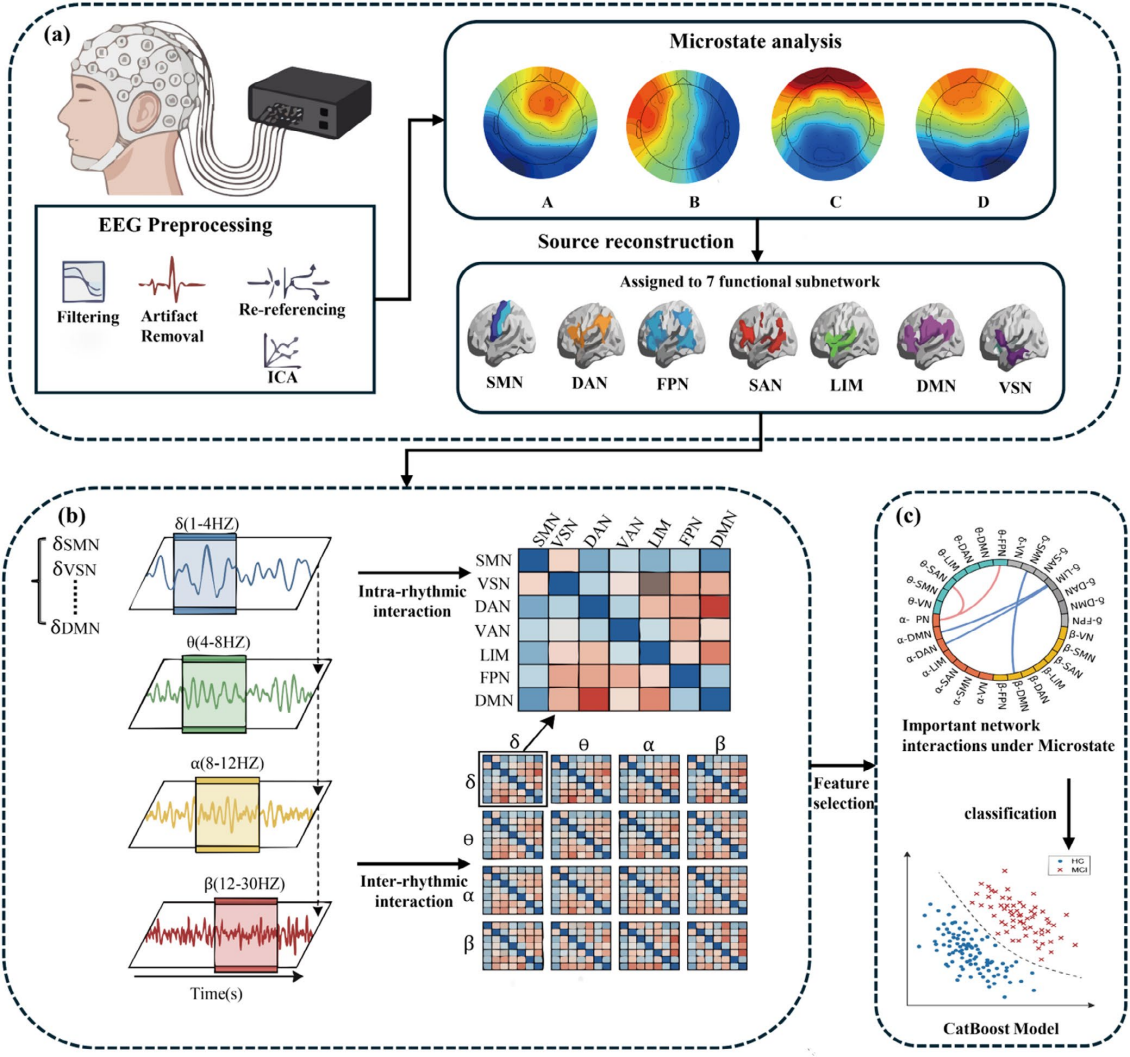
Illustration of the experimental pipeline. (a) Resting‐state data were recorded for each participant and, following preprocessing, underwent both microstate analysis and source reconstruction. (b) The *n:M* phase synchronization index was used to construct cross‐frequency network features within each microstate to analyze intra‐ and inter‐rhythmic interactions across the seven large‐scale networks. (c) The most important network interaction features were selected for classification.

Source reconstruction used weighted minimum norm estimation (wMNE) in BrainStorm [28], with depth weighting to reduce localization bias (Figure 1a). The forward model was computed with a three‐layer boundary element method in OpenMEEG [29] using the ICBM152 template [30] and standard conductivities (scalp/brain 0.33 S/m; skull 0.0066 S/m) [31]. Sources were constrained to the cortical surface, with dipoles oriented perpendicularly to the cortex. The wMNE inverse solution calculated current density on a 15,002‐vertex mesh, subsequently parcellated into 100 Schaefer‐2018 ROIs [32]. ROI activity was defined as the mean current source density across vertices, and ROIs were assigned to seven networks (VSN, SMN, DAN, SAN, LIM, FPN, DMN) to examine network‐specific dynamics and inter‐network coupling.

### 2.5 Construction of Microstate‐Specific Cross‐Frequency Coupling Networks

The construction of microstate‐specific cross‐frequency coupling networks involved several sequential steps. Initially, each participant's 5‐min EEG data, comprising 150,000 samples at 500 Hz, underwent labeling into four distinct microstate classes (A–D). This classification was achieved by determining the maximum spatial correlation with predefined template maps. Subsequently, for each microstate class, the time points corresponding to that state were isolated to generate a unique microstate‐specific time series. It is worth noting that the lengths of these segments varied among participants, reflecting individual differences in state duration. Following this, source reconstruction was performed, and each microstate‐specific series was then mapped to the seven large‐scale networks.

Signals from the seven networks were bandpass filtered into four sub‐bands: delta (1–4 Hz), theta (4–8 Hz), alpha (8–12 Hz), and beta (12–30 Hz) [33], using a bidirectional zero‐phase FIR filter (EEGLAB pop_eegfiltnew, MATLAB). Gamma was omitted because it is highly prone to muscle artifacts in resting EEG, which can bias coupling estimates [34]. The workflow is presented in Figure 1.

To quantify microstate‐dependent variability in MCI, we divided each network signal into non‐overlapping 5 s epochs. This allowed for n:m PSI analysis within each participant and microstate, yielding global and nodal graph metrics specific to each microstate (Figure 1b). These features were then used to classify individuals with MCI versus controls and to evaluate the Microstate‐specific Cross‐Frequency Coupling Network (MCFCN) model. This methodology provided insight into microstate‐specific information flow and elucidated network topology.

Our subsequent step involved exploring phase coupling, both within and between different frequency bands, across the seven large‐scale networks; the following computations were conducted for each 5‐s epoch:

The instantaneous phase was calculated using the Hilbert transform, defined as:

(3) $$\tilde{x}\left(t\right)=\frac{1}{\pi }\mathrm{PV}\int_{-x}^{\infty }\frac{x\left(\tau \right)}{t-\tau }\mathrm{d\tau}$$

where PV denotes the Cauchy principal value. The instantaneous amplitude $A\left(t\right)$ and instantaneous phase $\phi \left(t\right)$ were then derived as:

(4) $$A\left(t\right)=\sqrt{{\left[x\left(t\right)\right]}^{2}+{\left[\tilde{x}\left(t\right)\right]}^{2}}\&\phi \left(t\right)=\arctan\frac{\tilde{x}\left(t\right)}{x\left(t\right)}$$

Subsequently, the *n:m* Phase Synchronization Index (*n:m* PSI) was computed to quantify phase coupling between signals $x_{f_{m}}$ and $y_{f_{n}}$, with band center frequencies $f_{m}$ and $f_{n}$ over window *T*:

(5) $$\mathrm{PSI}\left(x_{f_{m}},y_{f_{n}}\right)=\left|\frac{1}{T}\sum_{t=1}^{T}e^{j\left[\Delta \phi \left(x_{f_{m}},y_{f_{n}},t\right)\right]}\right|$$

where the PSI ranges from 0 to 1, and $\Delta \phi \left(x_{f_{m}},y_{f_{m}},t\right)$ denotes the instantaneous phase difference, defined as:

(6) $$\Delta \phi \left(x_{f_{m}},y_{f_{n}},t\right)=n\cdot \phi \left(x_{f_{m}},t\right)-m\cdot \phi \left(y_{f_{n}},t\right)$$

For cross‐frequency coupling, n and m are the smallest integers satisfying the target frequency ratio [35]. In our data, delta (2.5 Hz center) and alpha (10 Hz center) yield *n:m* = 1:4, so the delta phase is scaled fourfold to align with the alpha phase. The n:m PSI reduces to within‐frequency phase synchronization when *n:m* = 1:1, commonly quantified by phase‐locking value. While within‐frequency coupling is extensively studied, this paper focuses on cross‐frequency effects. Specifically, n:m PSI quantifies the phase synchronization between oscillations at distinct frequencies, thereby reflecting interactions among neuronal assemblies within large‐scale networks.

### 2.6 Feature Selection and Classification

**FIGURE 2:**
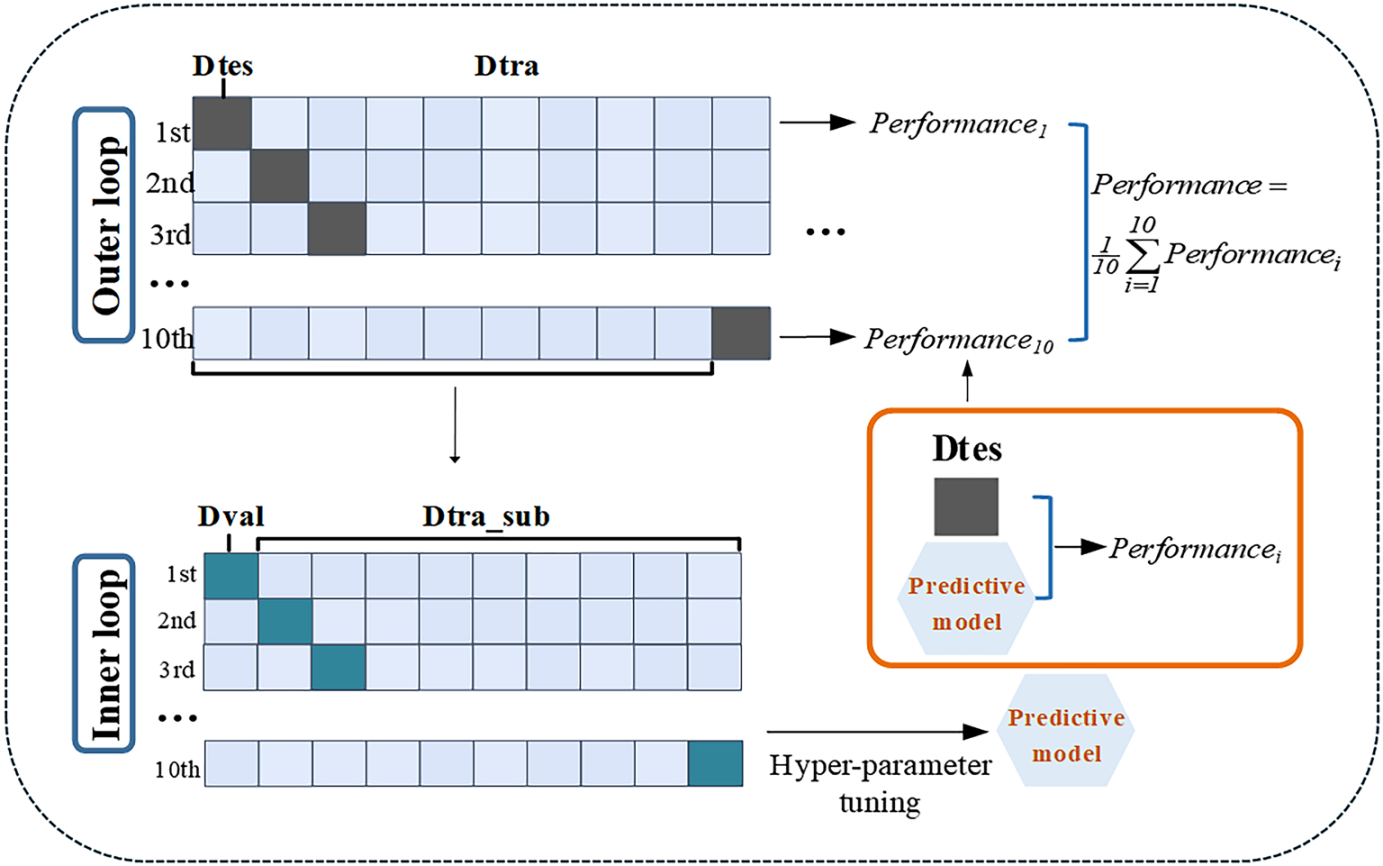
A 10‐fold nested cross‐validation (CV) scheme was utilized to estimate the weights and hyperparameters of the predictive model. The outer loop partitions the data into training and test sets, which are reserved for the final performance evaluation. The inner loop, operating exclusively on the outer loop's training set, is utilized for model selection and hyperparameter tuning. **Dtra** denotes the outer‐loop training set, **Dtes** the corresponding outer‐loop test set, **Dtra_sub** the inner‐loop training subset derived from **Dtra**, and **Dval** the inner‐loop validation set used for hyperparameter tuning. In each training fold, the mRMR algorithm was employed to determine a feature subset with maximum predictive relevance and minimum internal redundancy.

To assess whether MCFCN connectivity differentiates individuals with MCI from control subjects, we computed PSI‐based connectivity matrices for each microstate. From each microstate's 28 × 28 network, we extracted 378 unique cross‐frequency connections from the off‐diagonal upper‐triangular elements to serve as features. Classification was performed independently for each microstate, with the analysis restricted to participants who exhibited that specific microstate to ensure microstate‐specific validity. We employed a nested cross‐validation (CV) scheme for both hyperparameter tuning and performance estimation (Figure 2). In the outer 10‐fold loop, scikit‐learn Stratified Group K Fold [36] split subjects into a 9:1 training (**Dtra**) and test (**Dtes**) set, ensuring that data from the same participant did not appear in both sets. Within each **Dtra**, an inner 10‐fold CV further split data into a 9:1 training subset (**Dtra_sub**) and validation set (**Dval**) for model selection. Overall performance was reported as the mean across the 10 outer **Dtes** folds. In each training fold, the mRMR algorithm was utilized to identify a feature subset with maximum predictive relevance and minimum internal redundancy [37].

Classification utilized CatBoost, which mitigates gradient bias and prediction shift and is typically robust with limited tuning. Performance was evaluated by balanced accuracy (bACC), true positive rate (TPR), and true negative rate (TNR), which are informative under class imbalance. With TP, TN, FP, and FN denoting true/false positives and negatives, the metrics are defined as follows:

(7) $$\mathrm{TPR}=\frac{\mathrm{TP}}{TP+\mathrm{FN}}$$

(8) $$\mathrm{TRN}=\frac{\mathrm{TN}}{\mathrm{TN}+\mathrm{FP}}$$

(9) $$\text{bACC}=\frac{\mathrm{TPR}+\mathrm{TNR}}{2}$$

To confirm the robustness of the present findings, we expressed confidence intervals, assessed feature stability, and performed permutation testing. Confidence intervals (CIs) were estimated using a bootstrapping procedure with 1000 resamples. Condition labels (MCI vs. HC) were randomly shuffled across 1000 iterations. For each permutation, the entire nested cross‐validation framework was repeated to construct a null distribution for bACC. The empirical *p*‐value was then derived from the proportion of permutations that yielded a bACC equal to or greater than the original model's performance. For each feature f the mean importance $\left(\mu \_f\right)$ and standard deviation $\left(\sigma \_f\right)$ across cross‐validation folds were computed [38]. A stability score was defined as:

(10) $$\text{Stability}\left(f\right)=\mu \_f/\left(\sigma \_f+\varepsilon \right)$$

where ε is a small constant added to avoid division by zero.

Group differences in the retained stable features were analyzed using independent *t*‐tests or Wilcoxon rank‐sum tests, depending on Shapiro–Wilk normality results. To control for multiple comparisons, the Benjamini‐Hochberg False Discovery Rate (FDR) procedure was applied within each microstate, with significance set at an adjusted *p* < 0.05.

## 3 Results

### 3.1 Microstate Results

**FIGURE 3:**
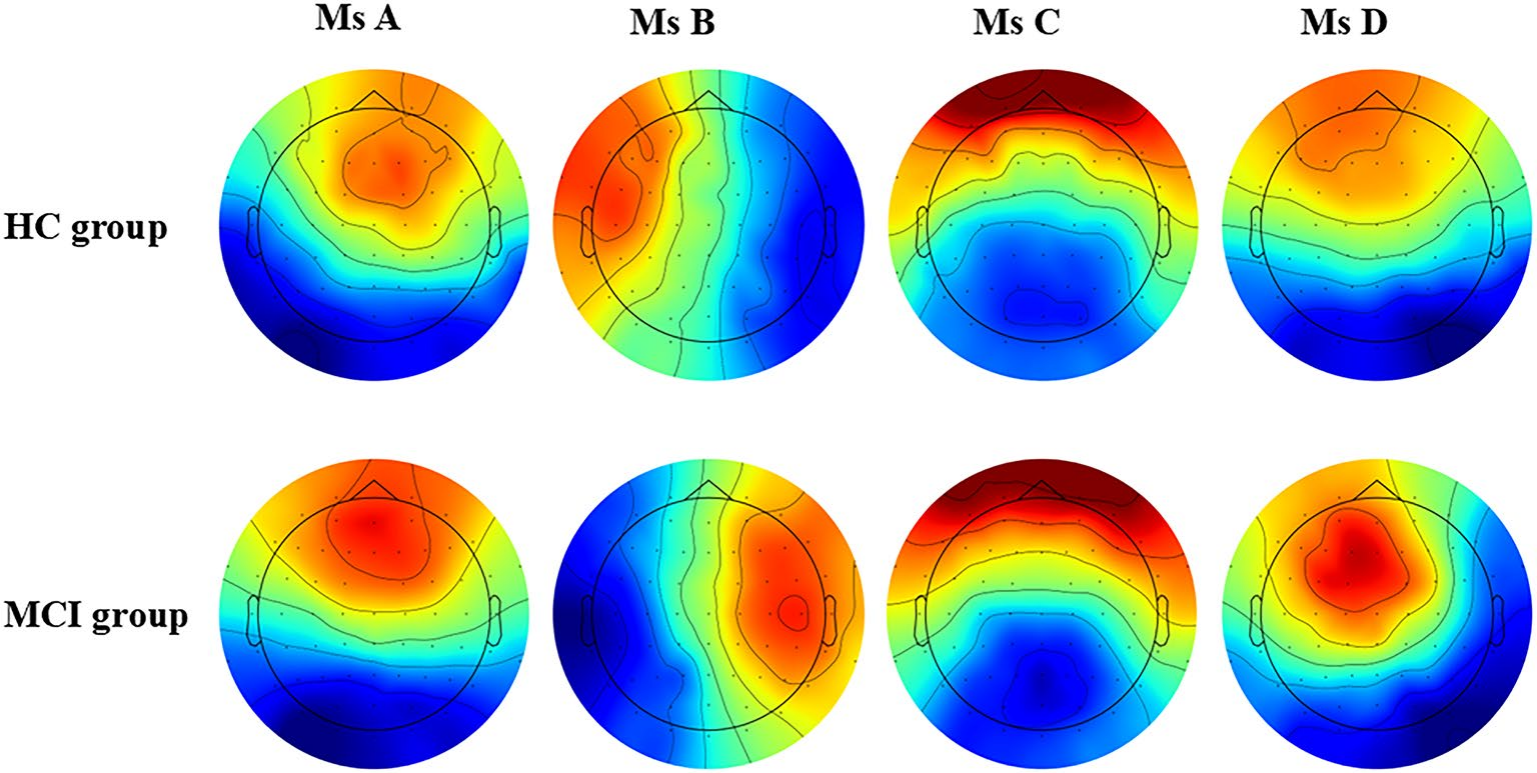
Comparison of the four canonical microstates between the HC and MCI groups. Ms, microstate.

Microstate segmentation revealed four stable topographic templates in both groups (Figure 3). These templates accounted for 78.57% of the variance in HC and 82.08% in MCI (*GEV*) and showed highly similar topographies across groups, indicating that a small set of quasi‐stable states effectively captures resting‐state brain dynamics. Specifically, Class A exhibited a left‐frontal to right‐posterior axis, while Class B displayed the opposite diagonal. Class C demonstrated an anterior–posterior polarity, and Class D showed a central symmetric pattern, all consistent with established canonical templates [21].

### 3.2 Feature Selection and Classification Results

**FIGURE 4:**
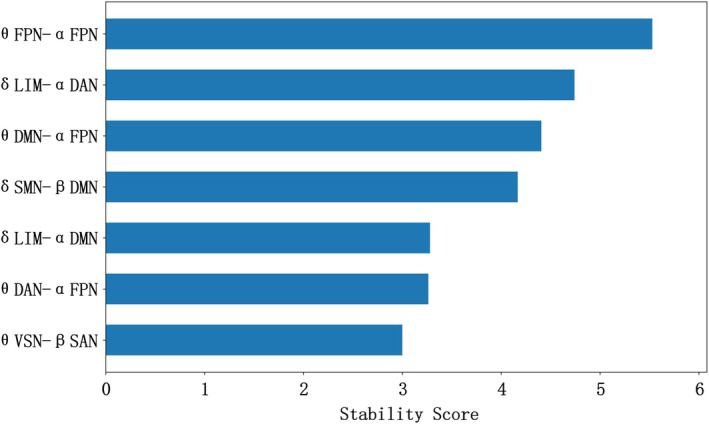
Stability scores of the EEG cross‐frequency features across cross‐validation folds in Microstate A.

After assessing feature stability and applying statistical filters, we identified significant cross‐frequency connections in Microstates A, C, and D. Microstate A, C, and D contained 4, 4, and 3 features, respectively. Microstate B was excluded due to a lack of significant connections. Figure 4 illustrates the feature stability scores for microstate A, obtained from the cross‐validation folds. As detailed in Table 2, the CatBoost classifier achieved optimal discriminative power in Microstate D within the nested cross‐validation framework. This model demonstrated a balanced accuracy of 84.4% (95% CI: 82.8%–86.4%), significantly exceeding chance levels (permutation *p* < 0.01, 1000 iterations). Similar statistical robustness was observed for models based on Microstate A (*p* = 0.011) and Microstate C (*p* = 0.016).

**TABLE 2:** Classification performance across different microstates (mean ± standard deviation) [95% CI].

Microstate	Accuracy (%) [95% CI]	Sensitivity (%) [95% CI]	Specificity (%) [95% CI]
A	84.1 ± 7.4 [79.5, 87.9]	85.5 ± 6.3 [81.9, 89.3]	82.8 ± 11.9 [75.1, 88.8]
C	82.5 ± 4.7 [79.7, 85.2]	83.5 ± 8.5 [78.2, 88.2]	81.5 ± 2.4 [80.0, 82.9]
D	84.4 ± 3.0 [82.8, 86.4]	81.0 ± 5.3 [78.1, 84.1]	87.8 ± 4.2 [85.4, 90.1]

### 3.3 The Most Significant Cross‐Frequency Interaction Networks

**FIGURE 5:**
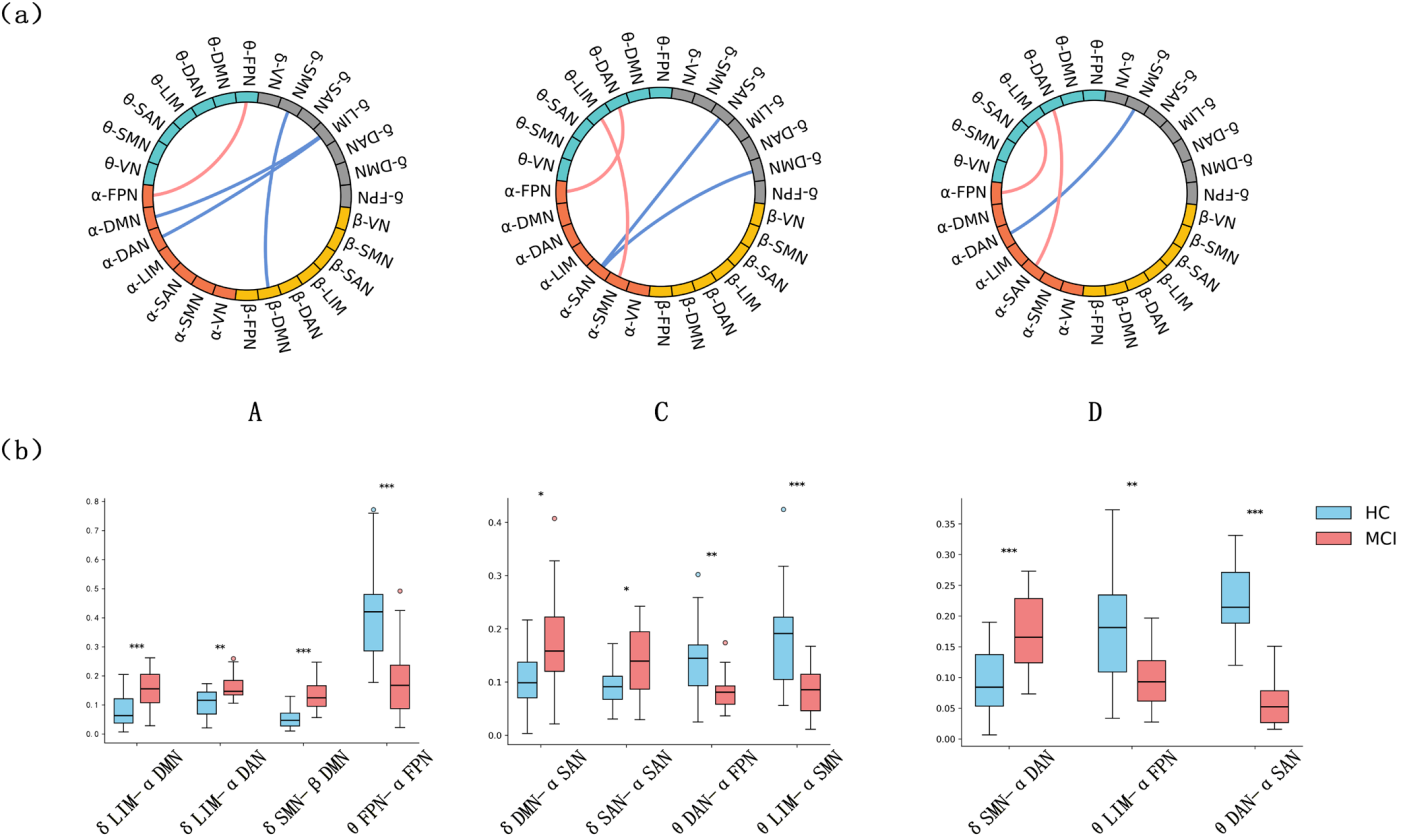
(a) Significant information interactions within the MCFCNs for microstates A, C, and D were observed between the HC and MCI groups. Solid blue lines indicate network connections that are significantly stronger in the MCI group compared to the HC group, while solid red lines denote connections that are weaker in the MCI group. (b) This panel illustrates group differences in connection strength. A statistical assessment of specific frequency‐network couplings across Microstates A, C, and D was performed. Blue and red boxes represent the HC and MCI groups, respectively, highlighting significant alterations in coupling dynamics within the MCI cohort. (*p < 0.05, **p < 0.01, ***p < 0.001).

Figure 5a illustrates the most consistently stable and significant features observed across all microstates. These features, clustered within Microstates A, C, and D, signify state‐specific network reorganization. In Microstate A, individuals with MCI demonstrated enhanced delta‐driven coupling, originating from the LIM and SMN, to alpha/beta activity within the DAN and DMN. Conversely, control subjects exhibited stronger theta‐alpha coupling exclusively within the FPN. Moving to Microstate C, MCI patients displayed increased delta‐driven coupling within the SAN and between the DMN and SAN. In contrast, control participants maintained stronger theta‐alpha integration, connecting the DAN and LIM with both the FPN and SMN. In Microstate D, connectivity was significantly elevated in controls (theta‐driven LIM‐FPN and DAN‐SAN links), with MCI showing only one increased connection (delta‐SMN to alpha‐DAN). Across the evaluated states, aberrant pathways frequently converged on theta‐LIM, alpha‐FPN/DAN, and delta‐SMN nodes, suggesting these regions might serve as primary hubs underlying disrupted spectral‐spatial coordination in MCI. Figure 5b displays the connectivity strength across distinct microstates.

## 4 Discussion

This study integrated EEG microstates with the n:m phase synchronization index to construct microstate‐specific cross‐frequency coupling networks, thereby investigating dynamic neural coordination in MCI. The findings revealed that MCI was associated with disrupted cross‐frequency phase coupling within Microstates A, C, and D. Furthermore, models incorporating these features achieved a balanced accuracy of 82.5%–84.4% in distinguishing individuals with MCI from healthy controls. Furthermore, cross‐frequency interaction networks involving regions such as theta‐LIM, alpha‐FPN, alpha‐DAN, and delta‐SMN were identified as core nodes characterizing MCI‐related neuropathological alterations. This spatiotemporal–spectral framework clarifies MCI neurophysiology and indicates EEG biomarkers with translational value for early detection.

Instead of isolating focal anatomical lesions, our framework is driven by pathological alterations across large‐scale functional networks. Because macroscopic network patterns are inherently robust to minor spatial inaccuracies [39], this design effectively accommodates the resolution constraints of standard EEG source reconstruction.

Recent studies have validated this approach, showing that standard head templates offer comparable diagnostic utility to individualized MRIs when classifying neurodegenerative diseases using macro‐scale features [40]. Therefore, the current spatial resolution is entirely adequate to support the cross‐frequency network dynamics captured by our model.

### 4.1 Consistency of Microstate Topography and Its Association With Disease Stage

Microstate topographies (A–D) were highly similar between HC and MCI, exhibiting a GEV of 78.57% and 82.08%, respectively. This supports the reliability of the templates and the proposition that microstates reflect instantaneous configurations of large‐scale brain networks [41]. The preserved topography also indicates early, selective impairment in MCI: unlike late‐stage AD, which shows marked microstate disorganization [42], MCI retains the basic spatiotemporal layout of core networks. Thus, these deficits likely reflect disrupted dynamic coordination rather than altered topography, which is consistent with the mild clinical phenotype in which daily function largely remains preserved [43]. This motivates our focus on abnormal cross‐frequency coupling within microstates. Microstates A (left‐frontal to right‐posterior), B (right‐frontal to left‐posterior), C (anterior–posterior), and D (central) matched canonical patterns, consistent with prior observations that microstate A is often linked to DMN, microstate C with DAN, and microstate D with SAN [44], although such mappings are not definitive. Despite similar topographies in HC and MCI, cross‐frequency coupling differed only in A, C, and D, and Microstate B did not enter the optimal feature set. This matches AD staging, in which primary visual cortex is relatively preserved until later disease [45, 46], supporting the biological specificity of the MCFCN approach. Taken together, these findings suggest a selective dysfunction in microstates associated with memory, attention, and executive control, thereby enabling microstate‐specific biomarker screening.

### 4.2 Cross‐Frequency Coupling Abnormalities Reveal Imbalance Between Network Integration and Segregation in MCI

We identified 11 significant cross‐frequency coupling features. Recurrent hubs included theta‐LIM, alpha‐FPN, alpha‐DAN, and delta‐SMN. These alterations, considering microstate‐specific functions, can be interpreted from three perspectives.

#### 4.2.1 Cross‐Frequency Coupling Abnormalities in Microstate A (DMN‐Dominant): Compensatory Memory Network Activity and Impaired Executive Function

Microstate A has been putatively mapped to the DMN, which supports memory and self‐referential processing [47]. In this state, MCI revealed stronger delta‐LIM to alpha‐DAN/alpha‐DMN coupling and delta‐SMN to beta‐DMN coupling, whereas controls demonstrated stronger theta‐FPN to alpha‐FPN coupling. The limbic system, including hippocampal–entorhinal regions, is an early site of tau pathology [48], and delta activity relates to plasticity during memory encoding [49]. Elevated LIM–DAN/DMN coupling in MCI may indicate compensation for reduced retrieval efficiency by strengthening slow–alpha coordination between memory and attention networks [50]. Moreover, an increase in delta‐SMN to beta‐DMN coupling indicates abnormal communication between sensory‐motor systems and the default mode network. This could potentially disrupt the natural separation of task‐negative and task‐positive networks when the brain is at rest. In controls, theta–alpha coupling within FPN likely facilitates working memory and executive integration by coordinating the maintenance and filtering of information [51]. This intra‐network cross‐frequency synchronization appears weakened in MCI, a change that may impair the executive control processes essential for effective memory organization and cognitive flexibility [52].

#### 4.2.2 Cross‐Frequency Coupling Abnormalities in Microstate C (Visuo‐Attentional Network‐Dominant): Decline in Visuospatial Integration Ability

Microstate C is generally considered to reflect coordinated activity of the VSN and DAN, though this association is approximate [53]. In this state, MCI showed stronger delta‐DMN to alpha‐SAN coupling and delta‐SAN to alpha‐SAN coupling, whereas controls revealed stronger theta‐DAN to alpha‐FPN and theta‐LIM to alpha‐SMN coupling. In individuals with MCI, the enhanced coupling between the DMN and the SAN, which functions as a critical network‐switching hub, might signal inefficient communication between these networks. This inefficiency could necessitate more robust slow‐to‐fast synchronization mechanisms to stabilize resting‐state network dynamics. Such findings align with previously documented visuospatial memory impairments observed in preclinical dementia [54]. Furthermore, heightened delta‐alpha coupling within the SAN suggests hyperexcitability of the salience network, potentially leading to excessive attentional capture by irrelevant stimuli. In controls, theta‐DAN to alpha‐FPN coupling supports visuospatial attention–executive coordination, facilitating top‐down information transfer and multisensory integration [55]. Theta‐LIM to alpha‐SMN coupling suggests tighter memory–sensorimotor integration, which may weaken in MCI and contribute to slower visuospatial processing and motor planning impairments.

#### 4.2.3 Cross‐Frequency Coupling Abnormalities in Microstate D (SAN‐Dominant): Disruption of Endogenous Cognitive Processing

Microstate D has been proposed to be linked to salience‐driven endogenous processing [56]. In this state, MCI showed increased delta‐SMN to alpha‐DAN coupling, whereas controls showed stronger theta‐LIM to alpha‐FPN coupling and theta‐DAN to alpha‐SAN coupling. The SMN supports sensory processing, while the DAN modulates goal‐directed attention. Elevated delta‐alpha coupling between the SMN and DAN in MCI may therefore indicate inefficient coordination between sensorimotor processing and attention. This could manifest as reduced efficiency in stimulus processing, aligning with the increased distractibility observed during the early stages of cognitive decline [57]. In control conditions, theta‐LIM to alpha‐FPN coupling underpins the integration of memory and executive functions. Concurrently, theta‐DAN to alpha‐SAN coupling plays a crucial role in endogenous attention control and the strategic allocation of resources based on salience among competing cognitive systems [58]. Disruptions to these fundamental cross‐frequency coordination patterns may underlie the global cognitive decline characteristic of MCI progression.

#### 4.2.4 The Central Role of Key Networks and Frequency Bands in the Pathophysiology of MCI: A Marker of “Network Coordination Disorder” in the Prodromal AD Stage

The recurrent involvement of theta‐LIM, alpha‐FPN, alpha‐DAN, and delta‐SMN indicates a nonrandom disruption of key oscillatory–network interactions in MCI. The limbic network, encompassing the hippocampus and entorhinal cortex, is affected early in AD, and theta disturbances are associated with episodic memory deficits [59]. Abnormal theta‐LIM coupling may indicate early synaptic dysfunction and diminished long‐range coordination. The FPN and DAN support are crucial for cognitive control and attention, relying on alpha activity for working memory and executive regulation. Consequently, altered alpha‐FPN/alpha‐DAN coupling might explain the impaired executive function and sustained attention observed in MCI.

**FIGURE 6:**
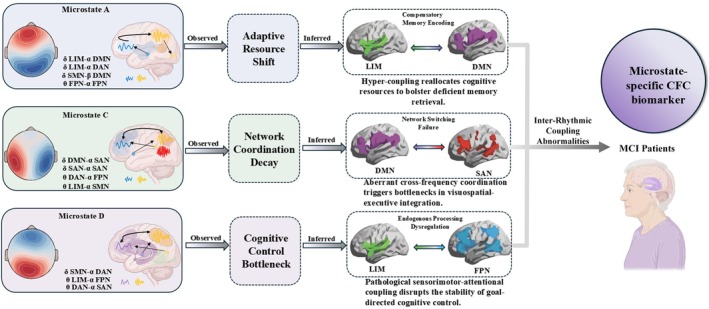
Predicted underlying mechanisms in MCI patients based on MCFCN. The schematic links observed electrophysiological markers (left) with inferred transitional states (middle) to demonstrate system‐level pathological consequences (right). Specifically, aberrant cross‐frequency coupling contributes to compensatory memory encoding during Microstate A, network switching failure in Microstate C, and endogenous processing dysregulation in Microstate D. Altogether, these distinct inter‐rhythmic coupling abnormalities serve as robust biomarkers for early MCI identification.

Delta activity in SMN relates to sensory integration and motor preparation [60]; its abnormal coupling may indicate inefficient sensorimotor processing or compensatory hyper‐synchronization early in disease. Figure 6 illustrates the proposed pathophysiological mechanism for microstate‐specific cross‐frequency coupling abnormalities in MCI. In the prodromal stage of AD, early tau accumulation and synaptic injury predominantly impact the limbic network, initiating a cascade of oscillatory dysregulation that subsequently propagates throughout large‐scale brain networks. This dysregulation presents as microstate‐dependent disruptions in cross‐frequency phase synchronization, characterized by abnormalities centered on theta‐LIM, alpha‐FPN/DAN, and delta‐SMN nodes. These network‐specific oscillatory impairments ultimately impair the spatiotemporal integration of neural information, contributing to the core cognitive deficits observed in MCI, such as episodic memory decline, attentional dysfunction, and executive impairment.

These cross‐frequency abnormalities suggest a cascade, originating from synaptic injury, that leads to large‐scale network dysregulation and cognitive decline [61]. They align with neuroimaging evidence of hippocampal atrophy, DMN disruption, and early tau pathology [62], indicating complementary electrophysiological, structural, and molecular signatures of prodromal AD. Thus, MCFCN features provide mechanistic EEG markers for MCI, strengthening multimodal biomarker models that link synaptic dysfunction to system‐level cognitive deficits and supporting integrated tools for early diagnosis and prognosis.

EEG microstates are increasingly recognized as electrophysiological fingerprints of large‐scale brain networks. However, the precise mapping between canonical microstate topographies (A–D) and specific resting‐state networks remains an approximation, not a definitive one‐to‐one correspondence. This mapping, derived from concurrent EEG‐fMRI studies and normative source localization analyses, suggests microstates A, B, C, and D are preferentially—but not exclusively—associated with the default mode network (DMN), visual network (VN), dorsal attention network (DAN), and salience network (SAN), respectively [63]. However, significant variability exists across studies, stemming from differences in preprocessing techniques, clustering algorithms, and the characteristics of subject populations. Therefore, throughout the present study, the interpretation of microstate‐specific coupling abnormalities as indicative of dysfunction in specific networks (e.g., DMN for microstate A) is a putative, theory‐guided inference. This framework serves to generate physiologically grounded hypotheses rather than to assert deterministic anatomical localizations.

### 4.3 Study Limitation

Our study integrated EEG microstates with n:m phase synchronization to identify aberrant dynamic network activity in individuals with MCI. The findings demonstrated microstate‐specific cross‐frequency reorganization, which supports the potential of these MCFCN features as promising EEG biomarkers. However, several limitations warrant consideration. Firstly, the recruitment of all participants from a single geographical region might restrict the generalizability of our findings. Secondly, the cross‐sectional nature of the study design only permits the observation of associations and does not enable us to ascertain whether MCFCN features can monitor disease progression or forecast the conversion to dementia. Consequently, longitudinal validation in larger, independent cohorts is essential.

Third, a limitation of the present study is the use of a generic template head model (e.g., MNI152) for EEG source reconstruction. Because this approach lacks individualized structural MRIs, it cannot account for subject‐specific anatomical differences such as skull thickness and cortical atrophy, which are common in MCI.

Consequently, this may introduce spatial localization errors of 10–20 mm [64] and potential spurious sources [65, 66], restricting our findings from being interpreted at a precise voxel‐wise level. In future, researchers should incorporate subject‐specific anatomy and digitized electrode positions, and consider multimodal integration with fMRI to improve the spatial interpretability of the findings. Fourth, our interpretation of the microstate‐specific findings hinges on the canonical, albeit approximate, mapping between microstate topographies (A–D) and large‐scale functional networks for example, the default mode, dorsal attention, and salience networks. While this mapping is widely employed and supported by multimodal evidence, its consistency across studies is not universal. Therefore, it should be viewed as a heuristic framework rather than a definitive atlas. Consequently, our network‐level interpretations, for example, associating microstate A with the DMN, are suggestive rather than definitive. Future research employing source‐localized EEG or simultaneous EEG‐fMRI is essential to directly validate these microstate–network correspondences in MCI.

Despite these limitations, the current framework provides a novel and physiologically interpretable EEG biomarker for early MCI identification, thereby laying the groundwork for future multimodal and longitudinal investigations.

## 5 Conclusion

Utilizing a microstate–cross‐frequency–network framework, we demonstrate that MCI exhibits abnormal cross‐frequency phase coupling within Microstates A, C, and D. These distinguishing features differentiated MCI patients from controls with a balanced accuracy of 82.5%–84.4% using CatBoost. Recurrent hubs—specifically theta‐LIM, alpha‐FPN, alpha‐DAN, and delta‐SMN—emerged as central markers of MCI‐related network alteration, which aligns with impaired coordination across memory, attention, and executive networks.

These findings elucidate MCI neurophysiology from an integrated spatiotemporal–spectral perspective and introduce a promising noninvasive EEG biomarker with significant translational potential. Furthermore, they advocate for the early diagnosis and intervention in Alzheimer's disease.

## Author Contributions

P.Z. and J.G.: study concept, design, and manuscript revision for important intellectual content. X.W., K.H. and L.C.: clinical data collection. L.G., X.Y.: EEG data analysis. C.J., L.H. and A.L.: EEG data collection. F.Y. and L.L.: manuscript writing. All authors read and approved the final manuscript.

## Funding

This work was supported by Science and Technology Program of XPCC (Grant Nos. 2023AB048 and 2023ZD033), the Fundamental Research Funds for the Central Universities of South‐Central Minzu University (Grant Nos. CZZ25009, CZZ24015, and PTZ25008), and by the 2025 Wuhan Municipal Key RD Program Projects (No. 2025061202030426).

## Ethics Statement

This study was approved by the Ethics Committee of the First Affiliated Hospital of Shihezi University (Approval No.: KJ2025‐332‐02).

## Consent

Written informed consent was obtained from participants.

## Conflicts of Interest

The authors declare no conflicts of interest.

## Data Availability

The datasets analyzed during the current study are not publicly available due to privacy/ethical restrictions but are available from the corresponding author on reasonable request.
